# Essential Oil of *Prangos ferulacea* (L.) Lindl.: Chemistry and Bioactivities

**DOI:** 10.3390/plants15020317

**Published:** 2026-01-21

**Authors:** Mijat Božović, Vanja Tadić, Milan Mladenović, Rino Ragno

**Affiliations:** 1 Faculty of Science and Mathematics, University of Montenegro, Džordža Vašingtona bb, 81000 Podgorica, Montenegro; 2 Institute of Medicinal Plants Research “Dr. Josif Pančić”, Tadeuša Koščuška 1, 11000 Belgrade, Serbia; vtadic@mocbilja.rs; 3 Kragujevac Center for Computational Biochemistry, Faculty of Science, University of Kragujevac, Radoja Domanovića 12, 34000 Kragujevac, Serbia; milan.mladenovic@pmf.kg.ac.rs; 4 Rome Center for Molecular Design, Department of Drug Chemistry and Technology, Sapienza University of Rome, Piazzale Aldo Moro 5, 00185 Rome, Italy; rino.ragno@uniroma1.it

**Keywords:** *Cachrys ferulacea*, volatile oil, phytochemicals, pharmacological properties

## Abstract

*Prangos ferulacea* (L.) Lindl. (Apiaceae) is an orophilous species with notable traditional uses, particularly across the broader Middle East region. Over the past 50 years, research on its essential oil has revealed the existence of several chemotypes. In addition to its chemical composition, there is also data on the biological activities of the essential oil. Among these activities, the most extensively studied are its antimicrobial and, to a lesser extent, antioxidant properties. Recent findings suggest the presence of additional biological effects, including cytotoxic, insecticidal, and phytotoxic effects. This review summarizes current knowledge and provides a foundation for future research, including more in-depth chemical and chemotaxonomic analyses, as well as exploration of the full therapeutic potential of this species.

## 1. Introduction

Plants have played a fundamental role in both traditional healing practices and modern drug discovery due to the diverse array of health-promoting substances they produce. In particular, a variety of secondary metabolites, including alkaloids, terpenoids, and phenolic compounds, have garnered significant attention for their therapeutic potential. While these compounds play vital ecological roles for the plants themselves, they also offer a wide range of pharmacological benefits for humans, manifesting through antioxidant, antimicrobial, anti-inflammatory, and anticancer activities [1]. In the search for novel natural products, plants belonging to the Apiaceae family (syn. Umbelliferae) have emerged as valuable sources of bioactive secondary metabolites [2]. This family includes approximately 3780 species grouped into around 444 genera [3]. It comprises many aromatic plants as well as economically important sources of vegetables, herbs, and spices. It is distributed nearly worldwide, but is most diverse in temperate zones of the Northern Hemisphere and at high altitudes in the tropics [4]. Notable genera, such as *Apium* L., *Foeniculum* Mill., *Coriandrum* L., *Daucus* L., and *Petroselinum* Hill, produce high levels of polyacetylenes, coumarins, and essential oils (EOs). Some of these compounds are unique to the Apiaceae family and are the subject of active phytochemical and pharmacological research. Due to their reactive nature, falcarinol-type polyacetylenes have been widely studied for their potent antifungal effects [5]. EOs derived from Apiaceae species exhibit strong antimicrobial and biocidal properties, making them attractive natural alternatives for use in integrated pest management systems [6]. Furthermore, a wide array of coumarins and flavonoids within the family exhibit varied antioxidant, anti-inflammatory, antiviral, and anticancer effects [7,8].

The genus *Prangos* Lindl. is considered one of the most notable in the Apiaceae family due to its rich profile of bioactive compounds and its long-standing role in traditional medicine in various regions, especially the Mediterranean and the Middle East [9]. Of particular relevance is *Prangos ferulacea* (L.) Lindl. (PF), which has been used for centuries in traditional medicine and is now receiving increased attention in modern research. Nevertheless, the existing literature is sparse and sometimes inconsistent, highlighting the need for a thorough examination of the available data. In light of this, the present review emphasizes the essential oil (EO) of this species, focusing particularly on the variability of its chemical composition and the range of its reported pharmacologically relevant biological activities. By consolidating current findings, this work aims to clarify the potential applications of the EO, while also considering the species’ broader therapeutic relevance.

## 2. The *Prangos* Lindl. Genus: Diagnostic Traits and Ethnobotanical Relevance

The genus *Prangos* Lindl., currently comprising 50 accepted species [3], has undergone significant taxonomic revision over the past few decades. Integrating molecular data and reassessing morphological features has not only reshaped the genus’s internal taxonomy, but has also led to the reclassification of species at the intersection between *Prangos* Lindl. and allied genera, such as *Cachrys* L. and *Bilacunaria* Pimenov & V.N.Tikhom. [10]. As a primary and widely used criterion in species-level taxonomy of the Apiaceae family, fruit morphological characteristics are also frequently employed to distinguish the genus *Prangos* Lindl. from related genera. In this context, the key criteria for generic differentiation include mesocarp division, the pattern of vascular bundle arrangement, and the presence of sclerenchyma. Thus, a two-layered mesocarp is a common characteristic of both *Prangos* and *Cachrys* species, with the former distinguished by vascular bundles diffusely arranged solely in the inner layer. In contrast, *Cachrys* species possess an additional sclerenchyma layer and vascular bundles within the outer mesocarp layer. *Bilacunaria* species lack mesocarp division but contain specific vascular bundles with two large lacunae [11].

Accurate comprehension of these taxonomic revisions is essential for advancing phytochemical and pharmacological research, as reliable species identification ensures reproducible results and scientific validity. These revisions have also redefined the geographic distribution of *Prangos* species, which now primarily covers the Mediterranean region, the Middle East, and parts of Central Asia [3]. Considerable intraspecific morphological variability has also been documented among different populations [12].

*Prangos* species have long been used in traditional medicine, particularly in Asian regions, due to their wide range of therapeutic properties. This highlights their considerable potential for broader pharmacological applications and underscores the need for further scientific investigation. Research has revealed that these species are rich in EO and contain various bioactive compounds, with coumarins, particularly furanocoumarins, being the predominant ones [9]. The most widely recognized indication for the use of these plants is the alleviation of gastrointestinal symptoms, although their wound-healing, tonic, diuretic, coagulant, antihypertensive, and aphrodisiac properties are also well documented [13,14]. Additionally, several species are traditionally associated with potential benefits in treating vitiligo, such as *P. pabularia* Lindl. [15]. Apart from their therapeutic uses, *Prangos* species are important sources of natural flavorings, spices, and food additives [9].

## 3. Morphology and Distribution of *Prangos ferulacea* (L.) Lindl., with Notes on Traditional Uses

**Figure 1:**
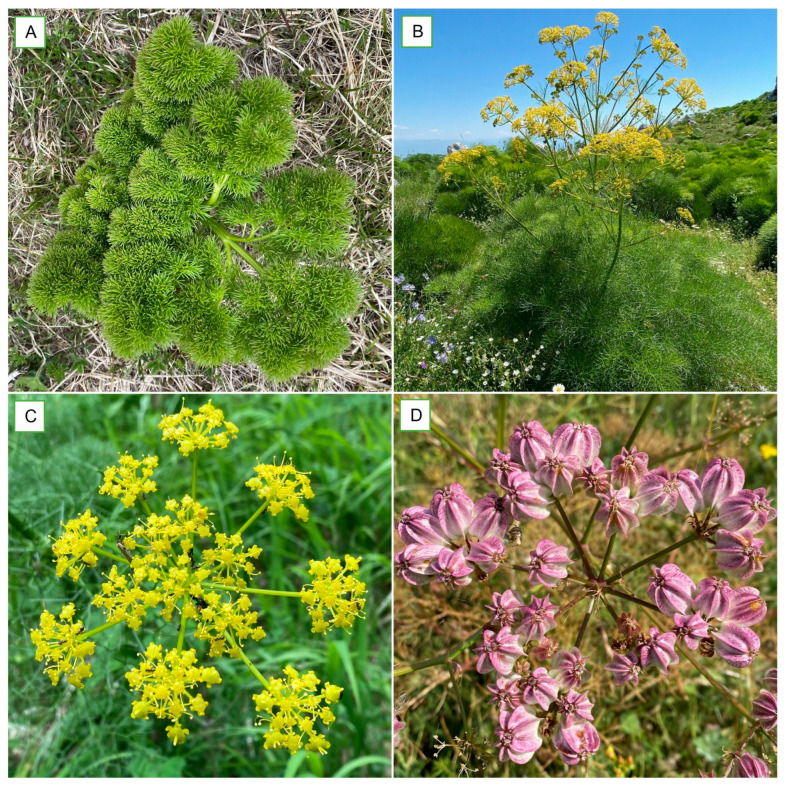
*Prangos ferulacea* (L.) Lindl.: young shoots (**A**), habitus (**B**), flowering (**C**) and fruiting umbels (**D**).

PF (*Cachrys ferulacea* (L.) Calest. and *Laserpitium ferulaceum* L., among the most common synonyms) is a robust, nearly glabrous perennial herbaceous plant reaching up to 180 cm in height. It develops a thick, fibrous rootstock and produces solid, striate stems that branch corymbosely in the upper part. The large basal leaves are 3- to 6-pinnate, up to 80 cm long, and have narrow, linear or filiform segments ranging from 10 to 45 mm in length. The margins are often slightly rough or puberulent. The upper leaves are smaller and less divided. They sheath the stem with broad, grooved bases. The inflorescences are compound umbels comprising six to eighteen robust rays accompanied by several linear-lanceolate, acuminate bracts and bracteoles that are membranous and caducous. The fruits (schizocarp composed of 2 mericarps) measure 10–30 mm in length and 10–15 mm in width. They are corky in texture, vary in shape from elliptical to globose, and are slightly laterally flattened, with faint ridges or slight lateral extensions, often displaying a whitish to pinkish hue (Figure 1) [16].

The genus *Prangos* Lindl. comprises xerophilous species with the greatest diversity occurring in the Irano-Turanian phytogeographic region [17]. PF is the type species of the genus and its native range extends across temperate regions from Southeastern Europe (including the Balkans and Sicily) through the Middle East to parts of Iran and the Caucasus [3,18]. The species exhibits a fragmented distribution across the Balkan Peninsula, predominantly occurring in mountainous regions between 1000 and 2000 m above sea level (a.s.l.), particularly in the southeastern Dinaric Alps, the Scardo-Pindic mountain range, and the northern Peloponnese. In contrast, at the westernmost (Croatia) and easternmost (Bulgaria) edges of its range, PF is confined to lower altitudes [19]. It typically occupies sunny, rocky habitats with a preference for alkaline soils and limestone substrates; however, occurrences on serpentinite have also been recorded in Albania [20]. PF primarily inhabits arid, open environments, such as calcareous slopes and dry meadows, often at higher elevations characterized by limited rainfall and nutrient-poor soils. These ecological preferences highlight the species’ strong adaptation to harsh, mountainous environments [21]. Interestingly, a population of this species has been recorded in North Macedonia growing on siliceous geological substrates [19], with similar occurrences documented in Sicily [22,23]. The species exhibits a relatively short phenological cycle, beginning with shoot emergence in late March to early April. It undergoes a distinct vegetative growth phase that culminates by mid-May, followed by the reproductive stage marked by flowering throughout June. Fruit development and maturation occur from July to early August, coinciding with the onset of senescence. By early August, the plant completes its annual life cycle and enters full dormancy. At this point, all aerial parts have senesced and are no longer visible above the soil surface [24,25].

PF has a long history of medicinal use, dating back to De Materia Medica by Dioscorides, in which it was referred to as “Ippomarathon.” It was used to treat kidney and urinary tract disorders, reflecting its ethnopharmacological importance [26]. Avicenna also mentions it in his Canon of Medicine as being suitable for dry-tempered individuals, moist wounds, and the elderly [27]. Its healing properties remain significant today, especially in northwestern Iran, where fresh, oily root exudate is traditionally applied to support recovery. These properties have since been experimentally confirmed [14]. Some authors have proposed that this species is the ancient silphion (or *Silphium*), a highly valued plant in Greco-Roman period known for its various uses that is now considered extinct. It is associated with the botanical representation on coins from Cyrene [28,29]. However, its chemical profile and broad Mediterranean distribution differ from the distinct characteristics and potent medicinal properties traditionally ascribed to silphion [30].

This plant is widespread in Sicily and predominantly grows on carbonate-rich mountains. It is a regular component of grazing areas for livestock, particularly cattle and sheep. Consumption of this plant by animals influences the organoleptic properties of their milk, lending a characteristic taste and aroma to local dairy products, such as traditional cheeses and salted ricotta, which are highly esteemed by the regional population [31]. Similar applications have been reported in Türkiye, where the plant is called “heliz” and used to make the traditional cheese otlu peynir. This cheese has a unique aroma, appearance, and antimicrobial properties [32,33]. Additionally, in mountainous regions, the plant is also called “çaşir” (or “caksir”) and is consumed as a vegetable, typically boiled, fried, or pickled [34,35]. Traditionally, it has been used as a stimulant decoction [36,37], as well as for managing digestive issues, hyperglycemia, and hypertension [38]. In Persian folk medicine, the plant is known as “jashir” (also “djashir” or “jooshir”) and is valued for its gastroprotective and hepatotonic effects. It is often used as a carminative with additional pharmacological properties, such as anti-flatulent, anti-inflammatory, antihelminthic, and broad-spectrum antimicrobial activity against fungi, bacteria, and viruses [24]. Its applications include laxative and sedative effects [39], and it is also used to treat infestations of external parasites in ruminant livestock [40]. In Serbia and neighboring regions, the plant’s root is commonly used in traditional herbal medicine to heal intestinal wounds, particularly hemorrhoids [41]. Beyond medicinal uses, the plant is commonly added as a flavoring agent to food and yogurt-based dishes [9,42].

## 4. Essential Oil Yield

As might be expected, EO yield varies considerably across studies, as it is well known that this parameter is influenced by a multitude of factors. These include various external conditions such as climate, altitude, and soil type, as well as internal factors such as genotype, plant organ, and the phenophase at harvest [43,44]. Some studies report EO yields as low as 0.2% from the aerial parts [45], while others have recorded values reaching up to 2.3% [46,47]. This variability is undoubtedly affected by the plant’s developmental stage [48]. The only available report on stem EO yield indicates a value of 0.8% [49]. In contrast, the contents of root-derived EOs vary markedly, ranging from 0.2% [50] to 1.2% [14]. Similar yield ranges have been reported for leaves [49,51,52,53,54,55], flowers [49,51,55,56], and fruits [29,34,47,52,57,58,59,60]. A comprehensive study from Iran examined ten distinct populations of PF collected from a wide range of altitudes and ecological conditions [52]; the EO yields from the aerial parts harvested during the flowering period ranged from 0.54% to 1.55%. Additionally, this study included an organ-specific analysis of a population growing at 2300 m a.s.l., revealing the lowest yield in leaves during the vegetative stage (0.8%), while significantly higher values were recorded in flowers (2%) and fruits (3%).

## 5. Overview of the Essential Oil Composition of *Prangos ferulacea* (L.) Lindl.

Interest in the EO of PF (PFEO) dates back to the late 1960s in the former Soviet Union. A fruit sample of this species, along with several others from the *Prangos* genus, was collected in what is now the Syunik Province of Armenia and analyzed for the first time. Although the data were presented differently in comparison with subsequent research (specifically, as percentages of individual compounds within the monoterpene and sesquiterpene classes) and are therefore not directly comparable to later literature, those studies, representing the earliest known data [61,62], have nevertheless contributed significantly to the understanding of this species’ EO. Even in that initial research, *α*-pinene, camphene, and *β*-phellandrene were the predominant compounds within the monoterpene fraction (42%, 22%, and 12%, respectively, relative to the total monoterpenes), while *α*-himachalene and *γ*-cadinene were the most abundant within the sesquiterpene fraction (31.4% and 33%, respectively, relative to the total sesquiterpenes) [61]. Some of these compounds have also been identified as dominant constituents in more recent studies.

Over the course of more than half a century, a substantial body of data has been accumulated, providing far deeper insight into the chemical composition of PFEO. A review of the literature clearly shows that most of the available data concerns EOs of this species originating from Iran. A smaller portion of studies focus on samples from Türkiye, Italy, and Greece. Limited data is available from the Balkans, represented only by Montenegro in a single study. Table 1 and Table 2 summarize this extensive dataset in detail, while the following section highlights the main trends, chemotypes, and notable variations related to geographic origin, plant parts, and extraction methods.

Despite the complexity and heterogeneity of the existing literature, a recurrent chemical profile emerges in the EOs derived from different parts of PF consistently observed across different phenological stages and geographic regions. This chemotype is mainly characterized by elevated levels of *α*-pinene and/or *β*-pinene [45,67,68,74], which often occur alone or together, sometimes accounting for over 70% of the total oil [60]. These primary constituents are often found alongside *δ*-3-carene, *α*- and *β*-phellandrene, highlighting a monoterpene-rich profile largely composed of cyclic structures [47,63,68,71,72,76]. From a biosynthetic standpoint, the menthane skeleton, formed through the cyclization of geranyl diphosphate, serves as the structural core of the reactive menthyl cation. This cation can give rise to various monocyclic and bicyclic monoterpenes. Specifically, through 1,3-hydride shifts and subsequent rearrangements, this intermediate yields isomers of pinene, carene, and phellandrene via proton loss [77]. Variations in the relative proportions of these monoterpenes among samples indicate intra-chemotype diversity. In some cases, phellandrene isomers and/or *δ*-3-carene prevail [63,67,69]. This may imply the possible existence of distinct sub-chemotypes within this group. This chemotype primarily occurs during the pre-reproductive and flowering stages and is distributed across various regions of Iran, including central, western, eastern, and northwestern areas. Furthermore, its presence in multiple plant organs–root [14,50], leaf [55,64], flower [52,55,64], and fruit [47,52,60]—further emphasizes its broad anatomical distribution and supports the concept of a chemically stable, ecologically persistent, and geographically persistent chemotype within the species.

Notably, a chrysanthenyl acetate-rich profile has been identified in fruit samples collected from Iran [65], Italy [29] and Türkiye [58]. This monoterpene shares structural features with *δ*-3-carene and is biosynthesized from geranyl diphosphate via similar carbocation intermediates that form a [3.1.1] bicyclic ring system. Unlike *δ*-3-carene, which is produced by the deprotonation of the carenyl cation, the biosynthetic pathway of chrysanthenyl acetate involves a nucleophilic attack on the same intermediate, resulting in the formation of chrysanthenol. Chrysanthenol then undergoes enzymatic acetylation [78]. However, since chrysanthenyl acetate is not the predominant component (up to 26.5%), it is more appropriately considered a variant or sub-chemotype, particularly due to its frequent co-occurrence with *α*-pinene and limonene, each exceeding 15%. Moreover, significant amounts of trimethylbenzaldehyde isomers characterize this chemical profile, particularly the 2,3,6- and 2,3,4-isomers. The former is most abundant in the Turkish sample, reaching 66.6% [58], while the latter is present at notable levels in the Italian material [29]. Thus, this compound plays a key role in defining the chemotype associated with these localities.

Following the first chemotype, a second profile characterized by elevated amounts of terpinolene and *γ*-terpinene has been observed [67,70]. These monocyclic monoterpenes are typically accompanied by *β*-phellandrene and minor amounts of *α*-pinene. This profile is particularly prominent in samples from the Fars and Semnan provinces of Iran, as well as in Crete, Greece, primarily during the pre-reproductive stage. Biosynthetically, this chemotype follows similar pathways to the previous one. Further rearrangements of the menthyl cation (which is also termed the *α*-terpinyl cation) open alternative biosynthetic routes, particularly via a hydride shift leading to the formation of the terpinen-4-yl cation. This process involves replacing one tertiary carbocation with another and leads to the formation of *α*-terpinene, *γ*-terpinene, and the alcohol terpinen-4-ol (an isomer of *α*-terpineol). This expands the structural diversity of monoterpenes within the menthane framework [77]. Moreover, research conducted in Italy and Türkiye on different plant parts has revealed substantial quantities of these monoterpenes, even in fruits [34,57,59].

Another clearly defined chemotype is distinguished by *β*-ocimene as the dominant compound. This linear monoterpene exists mainly as two geometric isomers: the trans-isomer, designated as (*E*)-*β*-ocimene, and the cis-isomer, referred to as (*Z*)-*β*-ocimene. Both are synthesized by the enzyme *β*-ocimene synthase, which catalyzes the conversion of geranyl diphosphate into diphosphate and *β*-ocimene, primarily in the (*E*) form. (*Z*)-*β*-ocimene and other monoterpenes, such as myrcene, are produced in smaller amounts [79]. Accordingly, several studies have reported (*E*)-*β*-ocimene as an important constituent of PEEO [57,59,69,70]. However, in some cases, it defined the entire chemical profile (up to 28.2%), as observed in leaf EOs from the Fars Province of Iran, where it co-occurs with limonene [53,54]. Even higher levels, up to 43.1%, were detected in aerial parts collected in Sicily, where it was found alongside its *Z*-isomer (15.8%) [75]. Additionally, the *Z*-isomer has been identified in several other Italian samples. For example, a study conducted on fruit material from Umbria found its content to be 26.8%, accompanied by *γ*-terpinene (27.8%) and terpinen-4-ol (12.2%) [59]. Furthermore, a (*Z*)-*β*-ocimene-rich variant has been identified in Sicilian samples. In these samples, (*Z*)-*β*-ocimene accounts for as much as 44.4% of flower oil and 64.9% of leaf oil. It is accompanied by a considerable amount of sabinene, suggesting possible stereochemical diversification within this chemotype [51]. It is worth noting that sabinene was also reported as the main constituent in the EO of fruits from a Sardinian population, reaching 15.9% [29].

Available data suggest that transitional chemotypes may also be present. These intermediate profiles are marked by varying proportions of the major monoterpene constituents that are typical of the described chemotypes and have been observed in numerous cases. Combinations involving key compounds like pinenes, *δ*-3-carene, *β*-phellandrene, and/or terpinene derivatives point toward a possible continuum of chemical variation rather than sharply defined categories [34,57,59,69,70]. For example, (*Z*)-ocimene (stereoisomer not specified) accounted for 36.3% of the flower EO from Iran (East Azerbaijan), where it co-occurred with *α*-pinene [60]. This variability likely reflects underlying biochemical plasticity influenced by genetic and/or environmental factors.

In addition to the previously described monoterpene-based chemotypes, analyses have identified a sesquiterpene-rich chemotype characterized primarily by *β*-caryophyllene and/or caryophyllene oxide. This chemotype is often accompanied by *α*-humulene and spathulenol [48,73]. The biosynthesis of these compounds originates from farnesyl diphosphate, the central precursor in sesquiterpene formation. Through enzyme-guided cyclization pathways, the precursor forms the humulyl cation. This cation can directly form humulene or rearrange into the caryophyllenyl cation. *β*-Caryophyllene is subsequently synthesized from the caryophyllenyl cation [77]. This chemotype is predominantly observed during the flowering and fruiting stages and has only been reported in the Kermanshah region of Iran.

Several other chemical profiles have been reported less commonly, each showing restricted organ specificity and/or regional occurrence. This highlights the chemical diversity and complexity within the species. Some Iranian studies have reported linalool-rich compositions (up to 36.7%) with notable amounts of caryophyllene oxide, 1,8-cineole, and *α*-pinene [49], while others have found limonene to be predominant (up to 55.1%) [66,67]. A distinct observation was reported in a study from the East Azerbaijan region of Iran, which revealed significant differences in EO composition before and during flowering, with (*E*)-anethole emerging as the principal constituent (reaching 95.5%) during flowering [45]. What makes the finding particularly unique is that phenylpropanoids (including anethole), although common in many Apiaceae species, have not been documented elsewhere for this species. In the material from Türkiye, a chemical profile dominated by *β*-elemene (up to 48.9%), along with prominent amounts of *α*-terpinolene and *β*-phellandrene, has been documented [34]. A chemically divergent variant, containing up to 85% of acetylenic 3,5-nonadiyne, has been reported exclusively from Montenegro [41]. This distinctive chemical peculiarity is notable, given the generally rare occurrence of this compound across plant species.

### 5.1. Sample Variability Across Plant Parts, Phenophases, and Extraction Methods

To date, research has been unevenly distributed across plant organs, with a strong emphasis on the aerial parts due to their significant ethnobotanical importance in traditional medicine and food preparation. Depending on the study, plant material was collected at different phenological stages, ranging from early vegetative growth to the reproductive period (Table 2). Regarding the stem, only two reports exist on EO analysis of this organ [49,63], and their results differ considerably. In contrast, leaves have been examined in a greater number of studies, with samples obtained during both the pre-reproductive phase and at flowering. Nevertheless, no significant differences in EO composition have been observed, regardless of these phenological variations (Table 1). Despite the root’s considerable importance in phytotherapeutic practice, particularly for wound healing, its chemical profile remains relatively unexplored. Two studies on Iranian material [14,50] reported monoterpene-rich oils, whereas the analysis of a Montenegrin root sample revealed a markedly different composition dominated by an acetylenic diyne compound [41]. Although the Iranian samples were collected during flowering and the Montenegrin sample during fruiting, this phenological difference is unlikely to account for such a marked divergence. Thus, the dominance of this acetylenic compound likely reflects inherent chemical specificity rather than phenophase-related variation. Reproductive organs have received considerable scientific attention (Table 1), leading to the identification of several chemotypes—particularly in fruits, where some unique profiles have been observed [34,58].

Most analyses were performed on air-dried plant material, though some studies included fresh samples [46,67,70,72]. While certain studies examined the impact of the state of the plant material on EO composition [46,67], no conclusive evidence suggests that this factor significantly alters the EO profile. Most studies used hydrodistillation, although steam distillation [47,48] and alternative extraction techniques, such as ultrasound-assisted [54], ohmic-assisted [53], and headspace solid-phase microextraction [33], have also been reported. Although extraction methodology is generally considered a factor that may influence EO composition, an analysis of the available published data on PFEO does not reveal consistent qualitative differences attributable to the extraction technique. The major constituents reported across studies are largely comparable, whereas variations are mainly observed in the relative abundance of individual compounds. As summarized in Table 1 and Table 2, the observed chemical variability among studies is therefore predominantly quantitative rather than qualitative. Nevertheless, due to heterogeneous experimental designs and limited direct comparability among studies, definitive conclusions regarding the specific impact of extraction methodology on PFEO composition cannot be drawn.

### 5.2. Shared Chemical Constituents Among Congeneric Prangos Taxa

The chemical complexity and diversity previously described for PF align with findings reported for other *Prangos* species. Monoterpenes (both hydrocarbon and oxygenated forms) generally represent the dominant constituent group in the EOs of these taxa [9,80]. Some of the key compounds defining the chemotypes of PFEO have likewise been identified as major components in the EOs of congeneric species, indicating a recurring chemical pattern across the genus. For example, the fruit EOs of *P. platychlaena* Boiss. and *P. uechtritzii* Boiss. & Hausskn. contain high concentrations of *α*-pinene (69.75% and 40.82%, respectively) [81]. This monoterpene is the most frequently encountered in EOs across species within the entire genus [80]. This trend is evident in *P. pabularia* Lindl., where it is found in high concentrations in both the aerial parts (32.4%) [37] and reproductive organs (33.87% in fruits and 21.46% in flowers) [82]. It is also present in *P. peucedanifolia* Fenzl (38.1% in the aerial parts) [37], *P*. *heyniae* H. Duman & M. F. Watson (44.8% in the roots) [83], and *P. uloptera* DC. (31.78% and 14.98% in fruits and flowers, respectively) [84]. The aerial parts of the latter also contain *δ*-3-carene (26.3%) [85]. On the contrary, a study on the aerial parts of *P. platychlaena* Boiss. reported *δ*-3-carene (9.25–43.17%), *α*-pinene (4.58–27.41%), *β*-pinene (3.72–25.55%), and *β*-phellandrene (4.02–17.88%) [86]. Likewise, *P. acaulis* (DC.) Bornm. [87] and *P. denticulata* Fisch. et Mey. [88] are characterized primarily by *δ*-3-carene, which is especially abundant in the roots of the latter. In addition to its notable *δ*-3-carene content [89], *P. asperula* Boiss. exhibits considerable levels of sabinene (29.8% in fruits and 20.6% in leaves) and *β*-phellandrene (19.2% in fruits and 19.0% in leaves) [90,91]. A considerable amount of sabinene was also found in the aerial parts of *P. meliocarpoides* Boiss. (16.7%) [92] as well as in the fruits of *P. denticulata* Fisch. et Mey. (26.1%) [88]. Although less frequently reported, *β*-ocimene has been identified in certain *Prangos* species. Specifically, (*E*)-*β*-ocimene was found in the EOs of the leaves, stems, and flowers of *P. platychlaena* Boiss. (25.93%, 22.94%, and 28.5%, respectively) [93], while the (*Z*)-isomer was detected in the aerial parts of *P. trifida* (Mill.) Herrnst. & Heyn (18.12%) [94].

Some *Prangos* species contain EOs that are rich in sesquiterpenes. For example, *β*-caryophyllene has been found in EOs of the aerial parts of *P. uloptera* DC. (17% and 27.1%) [72,95], *P. gaubae* (Bornm.) Herrnst. & Heyn (13.8%) [96] and *P. cheilanthifolia* Boiss. (16.1%) [97]. Its oxide has been reported in the aerial parts of *P. uloptera* DC. (15.9%) [95], *P. uechtritzii* Boiss. & Hausskn. (19.6%) [92] and *P. gaubae* (Bornm.) Herrnst. & Heyn (14.3%) [96]. Additionally, *P. corymbosa* Boiss. (40.7% in aerial parts) [98] and *P. pubularia* Lindl. (19.9% in fruits and 19.7% in flowers) [99], have been reported as rich sources of *β*-elemene.

The distribution of these shared dominant compounds across species may reflect common biosynthetic pathways, or might represent a case of adaptive convergence. Moreover, the recurrence of similar chemotypes could be relevant for biosystematic interpretations, aiding in the identification of natural groupings and the recognition of cryptic taxa within the genus *Prangos* Lindl. Even rare acetylenic compounds, not typically associated with PF [41], have been co-distilled with EOs in certain other *Prangos* species. Notably, 3,5-nonadiyne has been identified in the aerial parts (13.5%) [100] and fruits (24.5%) [101] of *P. platychlaena* Boiss. Moreover, several nonadiyne derivatives have been identified, including 3,5-nonadiyne-2-yl acetate (46%) in the aerial parts of *P. platychlaena* Boiss. [100] and (*Z*)-3,5-nonadiyne-7-ene (20.4%) in the root EO of *P. denticulata* Fisch. et Mey. [88]. Together, these findings highlight the broader chemical repertoire present across the genus, underscoring both its remarkable chemical diversity and the potential of even less common compounds as markers of phylogenetic or chemotaxonomic significance.

## 6. Current Insights into *Prangos ferulacea* (L.) Lindl. Essential Oil Bioactivities

Despite its widespread use in traditional phytotherapy, scientific evidence supporting the medicinal properties of PF, particularly those of its EO, remains limited. Considerable research has been devoted to solvent extracts [71,102], which are rich in phenolic compounds and coumarins known for their biological activity [103,104]. However, these findings cannot be directly applied to the EO, as its chemical composition and mechanisms of action differ significantly. Consequently, the biological properties of PFEO remain largely unexplored and warrant further targeted investigation.

Given the pronounced chemical variability of PFEO, including notable differences across various geographical regions, drawing definitive conclusions about its biological activities is challenging. The following sections present a comprehensive and thematically relevant synthesis of current findings regarding the bioactive potential of PFEO, with an emphasis on the major constituents identified in the examined samples. While the dominant compounds are considered key contributors to the observed effects, it is well-established that such bioactivities often result from multifaceted synergistic and antagonistic interactions inherent to the EO matrix [105].

### 6.1. Antimicrobial Activity

EOs are generally considered to exert antimicrobial effects by increasing membrane permeability and compromising the structural and functional integrity of microbial cell membranes. This ultimately leads to leakage of intracellular contents and cell death [105]. Direct-contact in vitro assays are most commonly used to evaluate this activity, including disk diffusion, well diffusion, and broth microdilution methods. The most commonly used quantitative indicator of antimicrobial potency is the minimum inhibitory concentration (MIC), which is typically determined by dilution methods. Despite their widespread use, these assays face challenges due to the oils’ hydrophobic and volatile nature. In diffusion assays, uneven migration of EO components through aqueous agar media can affect the outcome. In microdilution methods, solvents or emulsifiers (e.g., dimethyl sulfoxide [DMSO], ethanol) are often required to enhance solubility. These additives may influence microbial viability or interact with EO components, potentially compromising the reliability of results [106].

Of all the investigated bioactivities of PFEO, its antimicrobial potential has been studied the most thoroughly, with a focus primarily on antibacterial activity. While these studies offer valuable insights into its broad-spectrum activity, the efficacy of PFEO appears notably influenced by geographic variation in its chemical composition. Several studies have reported that PFEO is more effective against Gram-positive bacteria [51,53,54,65]. However, this trend is not consistently observed across all analyses. In some cases, even *Pseudomonas aeruginosa*, a Gram-negative bacterium known for its strong resistance to EOs [107], has shown unexpectedly high sensitivity [14,49]. Nevertheless, the generally higher susceptibility of Gram-positive bacteria to EOs is a well-established and extensively documented pattern in the literature, primarily attributed to structural differences in the bacterial cell envelope [106]. Gram-negative bacteria have an outer membrane that is rich in lipopolysaccharides. This membrane acts as a barrier against hydrophobic molecules, thereby reducing the permeability and efficacy of EO components. In contrast, Gram-positive bacteria lack this outer membrane, and their thicker but more permeable peptidoglycan layer allows easier diffusion of active compounds, facilitating membrane disruption and cell death [108,109]. These structural distinctions, along with variability in chemical composition, extraction methods, and test conditions, explain the differences observed among studies. Table 3 provides a detailed overview of all analyses and the complete set of results related to this plant species’ EO, as reported in the original studies. Data are grouped by the species of microorganism tested. In addition, the discussion below summarizes the main findings from each study, organized by testing method.

An in vitro study evaluated the antimicrobial potential of PFEO extracted from the roots of Iranian plant material collected in West Azerbaijan [14]. The study employed both agar disk diffusion and agar dilution methods to evaluate antimicrobial activity against a panel of standard bacterial and fungal species, including representatives of both the Gram-positive and Gram-negative groups, as well as *Candida albicans*. The EO exhibited marked efficacy against all tested strains at a concentration of 10 µg/disk, with inhibition zones (IZ) ranging from 14 mm (observed for *C. albicans* and *Escherichia coli*) to 21.5 mm (*Staphylococcus aureus*). In some cases, such as with *S. aureus*, *Staphylococcus epidermidis*, and *P. aeruginosa*, the EO’s activity surpassed that of amikacin, which served as the positive control. Determining the MIC values confirmed the oil’s broad-spectrum effectiveness, with the lowest MICs recorded at 5 µg/mL for *E. coli* and *C. albicans* and 10 µg/mL for *Salmonella paratyphi*. According to the authors, these effects are largely due to the high monoterpene content, particularly *β*-phellandrene and *δ*-3-carene.

Another study conducted on material from East Azerbaijan examined the antimicrobial activity of EOs extracted from the flowers and the fruits against a panel of microorganisms using the disk diffusion method [60]. *Bacillus cereus* was the most sensitive, with an IZ of 15 mm observed for both fruit and flower oils (oil quantity per disk not specified). Moderate inhibitory effects (9–12 mm) were recorded against the other pathogens. The authors attributed these effects to the high proportion of monoterpene hydrocarbons, particularly *α*-pinene, *β*-pinene, and (*Z*)-ocimene (no stereoisomer specified), which were identified as the major constituents of these oil samples. They suggest that the oils’ antimicrobial activity is primarily due to these abundant monoterpenes, which are known for their membrane-disruptive properties and broad antimicrobial potential.

A report from Tehran investigated the antibacterial activity of the fruit EO [65]. Using the disk diffusion method, IZ diameters of 12 to 14 mm were observed for *S. aureus*, *S. epidermidis*, and *E. coli*. Despite the high quantity of EO applied (2000 µg/disk), its antibacterial effect was less potent than that of neomycin, which was used as the control. Nevertheless, the MIC values, determined by the agar dilution method, ranged from 6.5 and 6.8 µg/mL (for *S. aureus* and *S. epidermidis*, respectively) to 12.5 µg/mL (*E. coli*), yet again higher than those of neomycin. The EO’s composition, notably high in chrysanthenyl acetate, limonene, and *α*-pinene, may underlie its promising antibacterial effects, particularly against *Staphylococcus* strains.

The EO extracted from the aerial parts collected in Lorestan Province was tested on several bacteria using the agar well diffusion method (40 μL/well) [74]. Depending on the strain, the oil (which belongs to the pinenes-rich chemotype, with *α*- and *β*-isomers comprising over 65% together) exhibited antibacterial activity with IZ diameters ranging from 8 mm (lowest sensitivity for *P. aeruginosa*) to 35 mm (highest sensitivity for *S. aureus*). The inhibitory effect was compared with gentamicin, the reference antibiotic, which produced IZ ranging from 12 to 20 mm. The larger IZ produced by the oil suggests promising antimicrobial potential, particularly against Gram-positive bacteria, a conclusion reached by the author.

Another study employed the well diffusion assay (50 µL/well) and found that PFEO from Zanjan (the plant part was not specified), demonstrated significant antibacterial activity against *Enterococcus faecalis*, with an IZ of 23 mm. This value was notably higher than that of gentamicin, used as the control drug (8 mm) [110]. The same study reported a MIC value of 2.27 μg/mL, as determined by the micro-broth dilution method.

Two studies conducted on leaf EO samples extracted from the plant material collected in Estahban [53,54] tested several bacteria. The oils demonstrated very weak antibacterial activity. In the disk diffusion assay, 10 µL of the oils produced IZ ranging from 10 to 16.8 mm. Tetracycline was used as the standard antibiotic for comparison, showing an IZ ranging from 14 to 22 mm. The MIC values determined by the broth microdilution method ranged from 4300 to 25,000 µg/mL, indicating low potency. The best effects were observed against the Gram-positive *Staphylococcus* and *Bacillus* strains, while *Salmonella typhimurium* and *Klebsiella aerogenes* exhibited the highest resistance. The EOs’ chemical composition was dominated by (*E*)-*β*-ocimene and limonene, which are compounds with limited antimicrobial activity [111,112,113]. This likely accounts for the weak inhibitory effects observed in these studies.

The EO isolated from the aerial parts collected in Shahmirzad demonstrated considerable antibacterial properties [69]. The EO was tested against a range of clinically relevant bacterial strains. Although it showed no activity against *B. cereus*, the MIC values, as measured by the agar well diffusion method (see Table 3 for details), were 8.19 µg/mL for *Staphylococcus saprophyticus* and 3.27 µg/mL for *E. coli*. This oil sample’s composition was characterized by a complex array of monoterpenes, dominated by *β*-phellandrene, *α*-terpinolene, *α*-pinene, *δ*-3-carene, and (*E*)-*β*-ocimene, indicating an intermediate chemotype.

An investigation on plant material from Esfahan examined the antibacterial activity against four bacterial strains using the broth microdilution method [49]. EOs extracted from the leaves, stems, and flowers of PF inhibited the growth of the tested bacteria to varying degrees, with MICs ranging from 0.0625 ppm to above 4 ppm. Among these, the leaf oil (rich in linalool, caryophyllene oxide, and *α*-pinene) demonstrated particularly strong antibacterial effects; its MIC values were 0.0625 ppm for *P. aeruginosa*, 0.25 ppm for *S. epidermidis*, 0.50 ppm for *S. aureus*, and 1.00 ppm for *B. cereus* (these values are reported in ppm and are approximately equivalent to µg/mL under the assay conditions). The stem and flower EOs also exhibited notable antibacterial potential. The stem oil had MICs of 0.5 ppm against *S. epidermidis* and *P. aeruginosa*, and the flower oil had MICs of 0.5 ppm against *S. aureus* and *B. cereus*. In addition to *α*-pinene, the stem and flower oils were particularly rich in 1,8-cineole, while the flower oil additionally contained significant amounts of linalool and lavandulyl acetate.

Another study evaluated the antifungal activity of the oil isolated from the aerial parts collected in Yasuj [72] against several clinical isolates of *Candida* species. The results showed remarkable antifungal efficacy, with the EO exhibiting even greater activity than the standard antifungal drug fluconazole. The MIC, determined using the microdilution method, ranged from 0.0097 to 0.0195 µL/mL, indicating a very strong inhibitory effect across multiple *Candida* strains. This oil sample was rich in both pinenes and *δ*-3-carene (together accounting for 70% of the oil). However, a significant amount of *β*-caryophyllene (17.7%) could have contributed to this notable activity.

Lastly, a study using plant material from East Azerbaijan analyzed the EO extracted from leaves during the flowering stage and revealed notable fungicidal activity [45]. The oil showed significant inhibition of mycelial growth at concentrations above 10 µg/mL when tested against the phytopathogen *Sclerotinia sclerotiorum*. Unlike the control group, in which fungal colonies occupied the entire Petri dish within seven days (approximately 45 mm in diameter), the presence of the EO restricted radial growth to 20–25 mm. This indicates a clear suppressive effect on fungal proliferation. The author attributed this pronounced antifungal activity mainly to the remarkably high (95.5%) content of (*E*)-anethole in the EO sample. This interpretation was supported by the EO extracted in the vegetative stage having reduced activity, as this phenylpropanoid was detected only in minor quantities (3.9%).

In addition to the aforementioned studies on Iranian plant material, a recent study examined the antibacterial properties of (*Z*)-*β*-ocimene-rich EOs extracted from the flowers and leaves of Italian-origin PF using vital cell counts and a broth microdilution assay [51]. Six reference strains were tested, and the results demonstrated dose-dependent activity. The flower oil exhibited activity against both Gram-positive and Gram-negative bacteria, while the leaf oil had a more pronounced effect on Gram-positive strains. *Bacillus* species were the most susceptible, with nearly complete inhibition at a concentration of 200 µg/mL for both oils. MIC values ranged from 100 to 200 μg/mL, or higher in some cases (*P. aeruginosa* and *S. aureus*), with Gram-positive bacilli being the most sensitive again. Considering the dominance of *β*-ocimene in the analyzed EO and the findings from previous research [53,54], it can be suggested that this compound has only a weak influence on antimicrobial activity. Indeed, several studies have investigated EOs rich in *β*-ocimene (particularly the *Z*-isomer) from other Apiaceae species, such as *Dicyclophora persica* Boiss. [114] and *Zeravschania membranacea* (Boiss.) Pimenov. [115], and have reported comparable or even weaker antimicrobial activity. Furthermore, the former study included testing *β*-ocimene separately as a pure compound, which resulted in considerably higher MIC values. This suggests that this monoterpene may play a more direct role in overall bioactivity.

### 6.2. Antioxidant Activity

The antioxidant capacity of plant extracts is commonly evaluated through various assays. Free radical scavenging capacity is usually determined using the 2,2-diphenyl-1-picrylhydrazyl (DPPH) and 2,2′-azino-bis(3-ethylbenzothiazoline-6-sulfonic acid) (ABTS) assays. The ferric reducing antioxidant power (FRAP) assay measures the ability of antioxidants to donate electrons and reduce ferric ions. Lipid peroxidation (LPO) assays examine the inhibition of oxidative damage to lipids. The scavenging capacity against hydrogen peroxide (H_2_O_2_), a reactive oxygen species, is commonly tested using the H_2_O_2_ assay. Antioxidant activities are often expressed relative to standard compounds, such as Trolox (a vitamin E analog), ascorbic acid, butylated hydroxyanisole (BHA), and butylated hydroxytoluene (BHT). Potency is frequently quantified using the IC_50_ value, which indicates the concentration needed to inhibit 50% of the radical or oxidative activity [116].

There are limited studies that have specifically investigated the antioxidant properties of PFEO. In contrast, the extracts of this species have attracted considerably more scientific attention. For example, the methanolic extract exhibited strong antioxidant activity [32]. Given the distinct chemical characteristics of EOs versus extracts, notably in phenolic compound levels, which are known to influence antioxidant capacity, direct comparisons of their antioxidant capacities seem inadequate. Below is a summary of the main observations regarding the antioxidant activity of PFEO.

In a study conducted on the Sicilian plant material of PF [51], the antioxidant potential of EOs derived from the flowers and leaves was assessed using several in vitro methods. Both oils demonstrated the ability to reduce reactive oxygen species (ROS) levels and increase the activity of key antioxidant enzymes (superoxide dismutase [SOD], catalase [CAT], and glutathione S-transferase [GST]) in polymorphonuclear neutrophils (PMNs) exposed to ozonide-induced oxidative stress. The EO from the flowers exhibited a stronger antioxidant response. Even at a concentration of 25 µg, ROS levels were significantly lowered, and at 100 µg, ROS levels were nearly fully normalized, reaching values comparable to those seen in non-stimulated control cells. Additionally, enzyme activity increased in a dose-dependent manner. The leaf oil reduced ROS levels most prominently at 100 µg and stimulated SOD and CAT activity at 50 and 100 µg; GST activity increased significantly at 50 µg. Regarding radical scavenging ability, the flower oil exhibited higher ABTS activity (IC_50_ = 100 µg/mL) than the leaf oil (IC_50_ = 500 µg/mL). However, the leaf oil performed slightly better in the H_2_O_2_ scavenging assay (IC_50_ = 50 µg/mL) than the oil from the flowers (IC_50_ = 60 µg/mL). In summary, both EOs demonstrated significant antioxidant activity; however, the oil derived from the flowers generally demonstrated greater efficacy across most of the conducted tests. The authors suggested that the observed differences in antioxidant capacity may be due to variations in their chemical composition. Specifically, they proposed that the higher antioxidant activity of the flower oil could be linked to the greater presence of compounds such as *γ*-terpinene, camphene, *α*-pinene, and limonene, which have previously been reported to have antioxidant properties [117,118]. Another study assessed the oil derived from the aerial parts of this Sicilian accession using three assays: DPPH, ABTS, and FRAP [75]. The results were expressed in terms of Trolox equivalent (TE) antioxidant capacity and IC_50_ values. The EO exhibited moderate antioxidant activity, with DPPH and ABTS values of 11.04 µmol TE/g and 60.64 µmol TE/g, respectively. The corresponding IC_50_ values were 726.5 µg/mL (DPPH) and 89.5 µg/mL (ABTS). In the FRAP assay, the oil demonstrated a TEAC value of 52.5 µmol TE/g.

The findings from both studies indicate that, while the EOs exhibit measurable antioxidant activity, it remains considerably lower than that of pure reference compounds, such as Trolox. This may not be entirely surprising, as similar results have been observed in *β*-ocimene-rich EOs from other plants, such as *Lindera glauca* (Siebold & Zucc.) Blume (Lauraceae) [119], *Ferulago angulata* (Schltdl.) Boiss. (Apiaceae) [120], as well as another *Prangos* species—*P. longistylis* (Boiss.) Pimenov & Kljuykov [121], where antioxidant capacity was characterized as weak to moderate. Conversely, certain studies have reported notable antioxidant effects of oil samples containing *β*-ocimene, such as those derived from *Tagetes erecta* L. (Asteraceae) [122]. These contrasting findings underscore the complex interactions between major and minor constituents that collectively determine the overall antioxidant potential of the EO.

A study on the EOs [56] extracted from the flowers and leaves of plants of Iranian origin aligns with the previously discussed findings. The antioxidant activity was evaluated using the ABTS and DPPH assays, with IC_50_ values of 8.01 µg/mL (flower oil) and 120.45 µg/mL (leaf oil) in the ABTS assay, and 23.90 µg/mL and 22.99 µg/mL, respectively, in the DPPH assay. These results indicate a moderate level of antioxidant activity. Both samples were found to be rich in *α*-pinene, particularly the leaf oil. The authors also noted that the antioxidant activity of these EOs was significantly lower than that of the extracts tested in the same study, some of which displayed even greater efficacy than BHT and ascorbic acid. Moreover, they emphasized the importance of phenolic and flavonoid content as key determinants of the enhanced antioxidant capacity observed in the extracts.

The antioxidant activity of the EOs isolated from another Iranian PF leaf sample was evaluated using the DPPH assay, demonstrating a dose-dependent but weak radical scavenging potential. In one study [53], hydrodistillation and ohmic-assisted hydrodistillation were used as extraction methods. The IC_50_ values obtained were 488.14 µg/mL and 570.52 µg/mL, respectively, confirming the weak antioxidant performance compared to BHT (IC_50_ of 17.34 µg/mL). The authors attributed the slightly superior activity of the sample obtained via hydrodistillation to higher levels of components such as terpinolene, *γ*-terpinene, and *α*-bisabolol, which are known for their hydroxyl functionalities and antioxidant properties [123]. A related study [54] further explored the antioxidant capacity of PFEO, this time using oils extracted via hydrodistillation and ultrasound-assisted hydrodistillation, continuing the investigation of method-dependent effects on oil composition and bioactivity. The IC_50_ values were 440 µg/mL and 160 µg/mL, respectively, indicating the ultrasound-assisted method’s greater efficacy. The authors attributed the higher antioxidant potential of the ultrasonic pretreatment to its enriched content of oxygenated compounds, such as aldehydes and hydroxylated terpenes, which generally exhibit greater activity in free radical neutralization. Nevertheless, compared to the reference antioxidants ascorbic acid (IC_50_ of 5 µg/mL) and BHA (IC_50_ of 18 µg/mL), the EOs displayed considerably lower potency.

### 6.3. Acetylcholinesterase Inhibitory Activity

In a recent study [75], the EO isolated from the aerial parts of PF collected in Sicily was evaluated for its in vitro acetylcholinesterase (AChE) inhibitory activity, using galantamine as a reference standard. The oil, rich in *β*-ocimene isomers, exhibited significant inhibition, with an IC_50_ value of 86.1 µg/mL compared to 11.2 µg/mL for galantamine. The authors attributed the observed activity primarily to the oil’s high monoterpene content (around 80%), referencing studies that highlight these compounds’ lipophilic nature, which allows them to readily cross cell membranes, including the blood–brain barrier. The authors also indicated the potential contribution of minor compounds, such as *α*-pinene, *β*-phellandrene, sabinene, and *p*-cymene, which have previously been reported as effective AChE inhibitors [124,125].

### 6.4. Cytotoxic Activity

The cytotoxic potential of the *β*-ocimene-rich EO obtained from the aerial parts of Sicilian origin was assessed in vitro using the 3-(4,5-dimethylthiazol-2-yl)-2,5-diphenyltetrazolium bromide (MTT) assay [75]. Three human cancer cell lines, including MDA-MB 231 (breast adenocarcinoma), HCT116 (colon carcinoma), and A375 (melanoma), were tested, and the oil exhibited moderate cytotoxic effects across all tested lines. The strongest growth inhibition was observed in the MDA-MB-231 cells (IC_50_ of 22.41 µg/mL), followed by the A375 cells (IC_50_ of 25.08 µg/mL), while the HCT116 cells showed the lowest sensitivity (IC_50_ of 30.35 µg/mL). Based on a previous study highlighting the anticancer properties of *β*-ocimene [126], the authors concluded that the observed cytotoxicity is likely attributable to this constituent, while also acknowledging the potential contribution of *α*-pinene and other minor components.

### 6.5. Insecticidal Activity

The fruit EO sample from Türkiye, rich in 2,3,6-trimethylbenzaldehyde and chrysanthenyl acetate, exhibited significant fumigant toxicity against the various developmental stages of the wasp *Trichogramma embryophagum* (Hymenoptera) and the moth *Ephestia kuehniella* (Lepidoptera) [58]. In that study, *T. embryophagum* showed greater overall sensitivity. The oil was most effective against the adult stage, achieving 100% mortality at concentrations of 1 µL/L air for *E. kuehniella* and 0.25 µL/L air for *T. embryophagum* after 24 h of exposure. Eggs and immature stages exhibited variable susceptibility. The LC_50_ and LC_99_ values for the larval stage of *E. kuehniella* were 379.662 and 538.755 µL/L air, respectively, while the values for the pupal stage of *T. embryophagum* were 5.947 and 19.568 µL/L air, respectively. Notably, *E. kuehniella* eggs were highly tolerant, with LC_50_ and LC_99_ values of 320.372 and 486.839 µL/L air, respectively, whereas *T. embryophagum* eggs were more sensitive than its pupae, with LC_50_ and LC_99_ values of 2.121 and 5.662 µL/L air, respectively. These differences in sensitivity among stages and species likely relate to structural features, such as chorion thickness and permeability [127]. In conclusion, the authors highlighted the stage-dependent fumigant efficacy of PFEO and its potential use as a botanical insecticide for controlling stored-product insects.

The larvicidal activity of the EO samples extracted from fruits, leaves, and stems of the Iranian plant material was assessed against third- and fourth-instar larvae of the mosquito species *Culex quinquefasciatus* and *Anopheles stephensi* (Diptera) [63]. According to the WHO protocol, groups of 25 larvae were exposed to five or more logarithmic concentrations of EOs for 24 h at 28 °C. Mortality rates were used to calculate lethal doses. The LC_50_ values for *C. quinquefasciatus* were 1.95 ppm for fruit oils, 1.76 ppm for leaf oils, and 2.06 ppm for stem oils, with corresponding LC_90_ values of 7.79, 7.26, and 6.87 ppm, respectively. For *A. stephensi*, the LC_50_ values were 24.21, 19.66, and 21.07 ppm, while the LC_90_ values were 70.99, 137.44, and 72.42 ppm, respectively. There were minor variations, but no statistically significant differences in larvicidal efficacy were observed among the different plant parts, particularly based on the LC_50_ values. This aligns with their similar chemical composition, which consists predominantly of phellandrenes and *α*-pinene. The authors suggest using PFEO as a potential insecticidal agent in integrated vector management programs targeting malaria and West Nile vectors.

### 6.6. Phytotoxic Activity

A study performed on the Iranian PF plant material (aerial parts) revealed two distinct EO chemical profiles: one rich in *α*-pinene during the pre-flowering stage, and another dominated by (*E*)-anethole during the flowering [45]. Both oils were tested for their phytotoxic potential using a lettuce (*Lactuca sativa* L. cv Varamin) seed bioassay. According to the results, the oil sample extracted during the reproductive phase exhibited stronger phytotoxic activity, significantly inhibiting root growth with an IC_50_ value of 244.19 mg/mL. A significant inhibitory effect on shoot length was observed only at the highest concentration (0.4 mg/mL) for both EOs, which reduced shoot length by approximately half compared to the control—from 17 mm to 8.8 mm for the vegetative-stage oil, and from 17.9 mm to 8.7 mm for the reproductive-stage oil. In contrast, the effect on radicle length was more pronounced. Although a reduction was also observed with the oil from the vegetative stage, the inhibition was considerably stronger with the flowering-stage oil. At 0.04 mg/mL, it caused about a twofold decrease in root length, while at 0.4 mg/mL, the reduction exceeded 2.5-fold (from 36.2 mm in the control to 13.2 mm). The authors suggested that this enhanced phytotoxicity may be related to the accumulation of (*E*)-anethole during the reproductive stage.

### 6.7. Muscle-Modulating Activity

A recent study used an in vitro isometric tension assay to evaluate the impact of Sicilian PFEO and its primary component, *β*-ocimene, on isolated rat duodenal and colonic muscle strips [128]. In the duodenum, the oil exhibited a biphasic effect: lower concentrations (12.5–50 μg/mL) induced contraction, while higher doses (100–200 μg/mL) triggered muscle relaxation. In the colon, the EO only exerted inhibitory effects, abolishing spontaneous contractions and maximally reducing muscle tone, particularly at 200 μg/mL. *β*-ocimene mirrored this inhibitory pattern in both intestinal regions in a concentration-dependent manner (7–120 μg/mL), suppressing spontaneous contractions while basal tone remained unaffected. The contractile response observed in the duodenum was abolished by ω-conotoxin, atropine, and neostigmine, suggesting involvement of enhanced cholinergic neurotransmission, likely through AChE inhibition. In contrast, the inhibitory actions of both the EO and *β*-ocimene were unaffected by inhibitors of nitric oxide (NO) signaling, K^+^ channels, and neuronal Ca^2+^ influx, indicating these pathways are not involved. Instead, both agents significantly reduced KCl-induced contractions and attenuated CaCl_2_ responses in Ca^2+^-free solution, consistent with the inhibition of extracellular Ca^2+^ influx via L-type voltage-gated Ca^2+^ channels. Additionally, they suppressed carbachol-induced contractions, indicating interference with intracellular Ca^2+^ release.

These findings underscore the dose-dependent and region-specific myomodulatory potential of PFEO. The authors suggest that its antispasmodic effect is likely driven by *β*-ocimene in synergy with other monoterpenes, such as *p*-cymene and carvacrol, via Ca^2+^ channel blockade. However, more research is needed to understand the mechanisms underlying its dual activity in the small intestine.

Another study was performed on the same EO to evaluate its modulation of uterine muscle contractility in non-pregnant rats and elucidate its underlying mechanisms of action [129]. The study clearly demonstrated that the EO exerts a potent, concentration-dependent inhibitory effect on uterine smooth muscle contractility in vitro. A key finding was the inhibition of spontaneous uterine contractions at concentrations ranging from 100 to 1000 µg/mL, with a reduction of approximately 76% at 300 µg/mL and complete suppression at 1000 µg/mL. These results confirmed the EO’s intrinsic spasmolytic potential and its relevance to conditions associated with uterine hyperactivity, even in the absence of external stimuli. The EO markedly reduced oxytocin- and KCl-induced contractions in a dose-dependent manner, achieving 96% and 89% inhibition at 1000 µg/mL, respectively. Since oxytocin mobilizes intracellular Ca^2+^ release through receptor-mediated signaling and KCl triggers Ca^2+^ influx via depolarization and L-type channel activation, the results imply interference with both pathways. Further experiments in a Ca^2+^-free medium with cumulative CaCl_2_ addition demonstrated that the oil significantly inhibited Ca^2+^-induced contraction at 1000 µg/mL, indicating blockade of L-type Ca^2+^ channels. The EO also suppressed contractions triggered by BaCl_2_ and oxytocin (agents that promote the release of Ca^2+^ from intracellular stores), suggesting interference with intracellular Ca^2+^ mobilization. To elucidate the contributions of individual EO components to the observed myorelaxant effect, the two major constituents, *β*-ocimene and carvacrol, were tested separately at concentrations of 3.6–120 μg/mL and 0.2–7.2 μg/mL, respectively. Both compounds exhibited tocolytic activity, reproducing the inhibitory effects observed with the whole EO and supporting their significant role in the overall antispasmodic effect.

Together, these results indicate that PFEO modulates uterine contractility via multiple targets within the Ca^2+^ signaling cascade, by inhibiting both extracellular Ca^2+^ entry and intracellular Ca^2+^ release. This dual mechanism likely underlies the EO’s strong antispasmodic effect and highlights its potential as a natural alternative to synthetic uterine relaxants, which are frequently associated with adverse effects. These findings provide a pharmacological basis for further investigating the PFEO’s potential in managing uterine dysmotility, menstrual pain, and related smooth muscle hyperactivity disorders.

### 6.8. Wound Healing Activity

In an in vitro study [14], the wound-healing properties of the PFEO distilled from roots collected in Iran were investigated. The EO sample was characterized by a high proportion of monoterpene hydrocarbons, with *β*-phellandrene and *δ*-3-carene identified as the major constituents. Biological activity was assessed using mouse L929 fibroblast cells. The MTT assay, which is based on 3-(4,5-dimethylthiazol-2-yl)-2,5-diphenyltetrazolium bromide, was used to evaluate cell viability and cytotoxicity. Fibroblast migration was examined through the scratch (wound-closure) assay, and extracellular matrix synthesis was measured with the Sircol type I collagen assay. At concentrations of 4 and 16 µg/mL, the oil accelerated fibroblast migration by up to 87% and increased collagen production in a dose-dependent manner without exhibiting cytotoxicity, even at 132 µg/mL. These effects were attributed to the EO’s high monoterpene content. The study concluded that the oil is a safe, promising topical agent capable of promoting fibroblast proliferation, migration, and extracellular matrix deposition—mechanistic processes that support its traditional application in wound care.

### 6.9. Antiparasitic Activity

A recent study [76] evaluated the effects of a PFEO sample rich in *β*-pinene and *δ*-3-carene on *Echinococcus granulosus* protoscoleces obtained from hydatid cysts under laboratory conditions. The oil was tested at concentrations of 0.625, 1.25, 2.5, 5, and 10 mg/mL, with exposure times of 3, 5, 10, 15, and 30 min. The most pronounced effect was observed at a concentration of 2.5 mg/mL after five minutes of exposure, resulting in 100% mortality of the protoscoleces. However, statistical analysis revealed that the combination of 5 mg/mL for 5 min was the most reliable and effective, representing the lowest concentration and shortest exposure time that consistently produced complete lethality. These results underscore the strong in vitro protoscolicidal effect of this EO and underscore the need for further in vivo and clinical studies to explore its therapeutic applications.

### 6.10. Bioactive Potential of Representative Constituents of Prangos ferulacea (L.) Lindl. Essential Oil

The chemotype-defining constituents of the PFEO, which have already been identified and discussed, are also recognized through experimentation for a variety of biological activities. It is well established that the interaction between the dominant and less abundant components of EOs often results in enhanced or complementary therapeutic effects that cannot be achieved by the individual components alone. Therefore, understanding the functional potential of representative major compounds offers valuable insight into the medicinal properties and broader biological applications of the EO. With this in mind, the following subsection will focus on the detailed pharmacological potential of selected key PFEO constituents.

Extensive research has demonstrated the diverse and potent biological activities of *α*- and *β*-pinene [130]. These structural isomers are known to exhibit potent antifungal effects against various pathogens, including *Candida*, *Aspergillus*, and *Trichophyton* species, as well as antibacterial activity against *S. aureus*, including MRSA strains [131]. Moreover, they have been shown to act synergistically with antibiotics to combat bacterial resistance [132,133]. These monoterpenes also possess antiviral properties and notably inhibit infectious bronchitis virus by interacting with viral nucleocapsid proteins, thereby preventing infection progression [134]. Their antioxidant properties have also been thoroughly investigated and reported [135]. *α*-Pinene demonstrates neuroprotective effects in models of ischemic stroke and epilepsy [136], as well as cardioprotective and anti-inflammatory effects [137,138]. It is also implicated in anti-aging, anxiolytic, and antiulcer activities [139,140]. Notably, *α*-pinene exhibits anticancer effects by inducing apoptosis and cell cycle arrest in various cancer cell lines [141,142] and by activating natural killer (NK) cells, thereby enhancing the potential of cancer immunotherapy [143]. *β*-pinene has been shown to enhance antitumor efficacy when co-administered with chemotherapeutic agents such as paclitaxel [142]. Additionally, *β*-pinene exhibits antihypertensive effects through endothelium-independent vasorelaxation by modulating Ca^2+^ influx [144]. Finally, pinenes not only inhibit insect development but also act as effective repellents [145,146].

Several pharmacological properties have been associated with *δ*-3-carene. Studies have shown that it has sedative effects and enhances sleep by modulating GABA_A_-benzodiazepine receptors [147]. It also exhibits antibacterial activity by disrupting the integrity of bacterial membranes and interfering with energy metabolism [148,149]. However, this monoterpene is considered to have only modest antioxidant potential [150,151]. In a murine model of allergic asthma, *δ*-3-carene demonstrated anti-inflammatory and anti-asthmatic effects by reducing interleukin expression and decreasing airway inflammation [152]. Nevertheless, it exhibited modest anti-inflammatory activity in the rat basophilic leukemia cell line, with effects less potent than those of pinenes [153]. Its insecticidal effects have also been confirmed, particularly through fumigant toxicity against storage pests, such as *Sitophilus zeamais* [145].

Due to its wide range of biological activities, *α*-phellandrene shows considerable promise for use in pharmaceutical, food, and agricultural applications [154]. It demonstrates antimicrobial activity against various bacteria and fungi, including *E. coli*, *S. aureus*, *P. aeruginosa*, and *C. albicans* [155], and inhibits the growth of *Penicillium cyclopium* by disrupting membrane integrity and causing ion leakage [156]. This monoterpene has been reported to enhance immune responses by promoting phagocytosis, increasing NK cell activity, and elevating T lymphocyte (T cell), monocyte, and macrophage levels in mice [157]. *α*-Phellandrene also induces cell cycle arrest and apoptosis in leukemia cells and exhibits in vitro cytotoxicity against melanoma and sarcoma cells, reducing tumor growth and nociception in vivo [154]. Studies have also shown that *α*-phellandrene provides pain relief by modulating multiple nervous system pathways and reducing inflammation, indicating its potential in treating chronic pain and neuropathic disorders [158]. It promotes wound healing by reducing inflammation and oxidative stress, and by stimulating fibroblast migration and collagen deposition, which helps with tissue repair [159]. Additionally, it demonstrates strong toxicity and repellent activity against agricultural and indoor pests, including *Anopheles quadrimaculatus*, *Aedes aegypti*, *Blattella germanica*, *Lucilia cuprina*, and *Bemisia tabaci* [160,161,162,163]. Although the *α*-isomer has been extensively studied, data on *β*-phellandrene remain limited. Only scattered reports associate *β*-phellandrene-containing EOs with antimicrobial activity, such as that of *Bursera morelensis* Ramírez (Burseraceae) [164], and with anti-inflammatory and cytotoxic effects observed in oils isolated from some *Pinus* species (Pinaceae) [165]. Nevertheless, one study demonstrated the DNA strand-breaking potential of *β*-phellandrene in a comet assay at high doses, suggesting possible genotoxic effects [166]. These findings highlight the need for more targeted studies on the pharmacological roles and mechanisms of *β*-phellandrene.

Chrysanthenyl acetate exhibits notable biological activities, including antimicrobial effects against *S. aureus*, *P. aeruginosa*, and *E. coli*. It has a strong ability to inhibit *P. aeruginosa* biofilm formation by about 65% at sub-inhibitory concentrations [167]. It also enhances the activity of key antioxidant enzymes (SOD, CAT, and glutathione peroxidase), suggesting direct and indirect antioxidant properties by promoting enzymatic defenses. Additionally, it shows no cytotoxicity on human keratinocytes, supporting its potential for safe therapeutic use. However, a study on the EO of *Asteriscus graveolens* (Forssk.) Less. (Asteraceae), which contains a significant amount of this compound, showed weak radical scavenging capacity, good reducing power, and strong antifungal effects, with up to 94.12% inhibition against *Fusarium culmorum*, as well as antibacterial activity, most pronounced against *B. cereus* [168]. The study also reported significant anticancer potential, revealing high cytotoxicity against human liver carcinoma and rat pheochromocytoma cell lines, surpassing the activity of the positive control. Additionally, evidence indicates that this monoterpenoid ester may exert analgesic and anti-migraine actions by interfering with prostaglandin pathways [169,170].

Several studies have reported the notable bioactivities of *γ*-terpinene and EOs containing it as a major compound. For instance, *Satureja thymbra* L. (Lamiaceae) oil exhibited strong antioxidant potential and significant antimicrobial effects against multiple strains, particularly fungi [171]. γ-Terpinene inhibits lipid peroxidation and acts as a natural antioxidant in edible oils [172]. Furthermore, studies on its synergistic antioxidant effects suggest that *γ*-terpinene may extend the shelf life of oils under high-temperature conditions [173]. When complexed with *β*-cyclodextrin, *γ*-terpinene demonstrated antihyperalgesic effects in a tumor-induced neuropathic pain model, effectively reducing mechanical hyperalgesia without impairing motor coordination [174]. *γ*-Terpinene was found to reduce leukocyte migration, edema formation, and enzyme activity, suggesting significant anti-inflammatory and antinociceptive effects in models of acute joint inflammation [175].

Terpinolene and terpinolene-rich EOs have exhibited multiple promising bioactivities. For instance, the oil of *Ferula macrocolea* (Boiss.) Boiss., predominantly (more than 70%) composed of terpinolene, was evaluated for its antileishmanial and cytotoxic effects against *Leishmania tropica* [176]. The study found that terpinolene exhibited stronger inhibitory effects on both promastigote and amastigote stages of the parasite, compared to the whole EO and the reference drug glucantime. Additionally, terpinolene induced a dose-dependent increase in NO production in macrophages, altered plasma membrane permeability, and markedly reduced infection rates by over 79%. Another study [177] showed that terpinolene had no direct antibacterial activity against *S. aureus* but reduced the MICs of oxacillin and ethidium bromide, suggesting inhibition of *β*-lactamase and efflux pumps. Similarly, terpinolene potentiated terbinafine activity against the dermatophytes *Microsporum canis* and *Trichophyton interdigitale* by promoting K^+^ efflux and compromising fungal membrane stability despite lacking strong intrinsic antifungal activity [178]. Notably, toxicity tests in *Drosophila melanogaster* revealed significant mortality and concentration-dependent locomotor impairments [177]. In a chronic inflammation model, terpinolene improved the efficacy of diclofenac synergistically by reducing hyperalgesia, edema, and inflammatory infiltration without inducing gastric lesions [179]. This effect appeared to involve serotonergic (5-HT2A) pathways. Other studies revealed that terpinolene downregulates AKT1 (a gene linked to cell survival) and suppresses proliferation in K562 leukemia cells, suggesting potential anticancer activity [180].

*β*-ocimene exhibits diverse bioactivities that may offer health benefits. As previously mentioned, data regarding its antimicrobial activity is inconsistent. While some *β*-ocimene-rich EOs have demonstrated promising antimicrobial effects [181,182], others have shown only limited or negligible activity [114,120]. Interestingly, the EO of *Astronium graveolens* Jacq. (Anacardiaceae), containing *β*-ocimene, *α*-pinene, *δ*-3-carene, and *α*-phellandrene as major constituents (though common in PF, this combination has not been observed), exhibited remarkably strong antibacterial activity, irrespective of bacterial species or resistance mechanisms, and comparable to the potent inhibitory effect of tigecycline [183]. Similarly, some reports indicate significant antioxidant activity associated with *β*-ocimene or oils containing it [184,185], yet other studies have found the activity to be weak [181,186]. Furthermore, this monoterpene possesses anti-inflammatory properties, with some studies demonstrating its ability to inhibit pro-inflammatory pathways, such as Toll-like receptor 4 and NOD-like receptor pyrin domain-containing 3, reducing cytokine levels and oxidative stress. It also blocks pyroptosis, lowering inflammation, joint damage, and pain in arthritis and ulcer models [187,188]. In addition, the EOs of *Oenanthe crocata* L. (Apiaceae) and *Isolona dewevrei* (De Wild. & T. Durand) Engl. & Diels (Annonaceae), both characterized by a high content of *β*-ocimene isomers, showed marked anti-inflammatory potential; the former reduced NO production and suppressed inducible NO synthase expression in activated macrophages [189], while the latter effectively inhibited lipoxygenase activity, with an IC_50_ value comparable to nordihydroguaiaretic acid (used as a reference) [190]. *β*-ocimene has also exhibited strong in vitro activity against *Leishmania amazonensis*, exerting direct and immunomodulatory effects at non-toxic concentrations to host macrophages, with a selectivity index greater than that of standard reference drugs [191]. Some research indicates that *β*-ocimene may have insect-repellent and fumigant properties, which could be exploited in agricultural practices to reduce pest damage [192,193].

The sesquiterpene *β*-caryophyllene and its oxygenated form possess a wide range of biological activities. The antimicrobial properties of *β*-caryophyllene have been observed against a broad spectrum of pathogens, with particularly strong activity toward *S. aureus* [194]. Additionally, it has been shown to inhibit the growth and virulence of *Helicobacter pylori* in both in vitro and in vivo models [195]. The oxide exhibits even broader and more consistent antimicrobial activity across Gram-positive and Gram-negative bacteria [196]. *β*-caryophyllene has notable anti-inflammatory activity by selectively activating the CB_2_ receptor and suppressing proinflammatory cytokine expression [197], as well as strong antioxidant activity [194], demonstrating superior inhibition of lipid peroxidation compared to reference antioxidants probucol and vitamin E, together with strong free radical scavenging of hydroxyl radicals and superoxide anions [198]. By reducing neuroinflammation and demyelination, which are key factors in neurodegenerative diseases, *β*-caryophyllene exhibits neuroprotective properties [199]. It also promotes wound healing by enhancing re-epithelialization and supporting tissue remodeling [200]. Furthermore, caryophyllene oxide acts synergistically with paracetamol to enhance the analgesic effect and contribute to gastric mucosal protection, highlighting its potential as a complementary anti-inflammatory and antioxidant agent [201]. *β*-Caryophyllene exhibits anticancer activity by selectively inhibiting cancer cell growth, inducing apoptosis through mitochondrial disruption and caspase activation, and enhancing the effectiveness of chemotherapy drugs. Its oxygenated form acts on key cancer-related signaling pathways for an even stronger effect [202]. While *β*-caryophyllene is generally considered safe at pharmacological levels, its inhibitory effect on cytochrome P450 enzymes, particularly with its oxide derivative, must be considered when co-administered with other medications [203].

## 7. Concluding Remarks and Future Perspectives

This review summarizes the available literature on the chemical composition of PFEO by consolidating data from various studies into a single, comprehensive source. Multiple chemotypes have been identified, some of which can be correlated with specific geographical regions. Considering the plant’s natural range, there is an evident lack of data from the Balkan countries (although it is sporadically distributed in these regions), where only a single sample was analyzed over 20 years ago. This gap may be attributed to the plant’s limited use in traditional phytotherapy in these areas. Nevertheless, that study [41] revealed a highly specific chemical profile, underscoring the need for further investigation, which could provide valuable insights from a chemotaxonomic perspective.

Alongside the analysis of the chemical composition, a review of studies on biological activities was also conducted. The collected data suggest promising antibacterial effects, demonstrated as strong in some cases [49,63,110], while the antioxidant activity appears to range from low to moderate. However, the prediction of bioactivities is complicated by well-known synergistic and antagonistic interactions among EO constituents, making it difficult to draw definitive conclusions from the current data. Moreover, the EO of this plant generally lacks monoterpene phenols (e.g., thymol and carvacrol) or phenolic phenylpropanoids (e.g., eugenol)—compounds commonly associated with stronger biological activities—which may partially explain the observed results. From an applied perspective, the absence of these constituents suggests that PFEO may be more suitable for applications requiring moderate and selective antimicrobial effects rather than broad-spectrum potency. Such properties may be advantageous in pharmaceutical or cosmetic formulations, as well as in food-related applications, where reduced irritation and lower toxicity are desirable.

There is limited and sporadic evidence pointing to additional potential activities, such as fumigant, phytotoxic, and anti-echinococcal effects. However, the currently available data are insufficient for firm conclusions. For example, the antifungal activity appears noteworthy, particularly against *Candida* species, despite being supported by only a few studies [69]. Additionally, studies on cytotoxicity, acetylcholinesterase inhibitory activity, and modulation of smooth muscle contractility have solely examined EO samples of the *β*-ocimene-rich chemotype. Similarly, the wound-healing properties widely recognized in traditional root-based applications have been demonstrated only for the chemotype rich in *β*-phellandrene and *δ*-3-carene. This emphasizes the need for further exploration of other chemical profiles. In this context, the reported fumigant and phytotoxic activities, although limited, indicate a potential role of PFEO or selected chemotypes in agricultural applications, particularly as environmentally friendly alternatives to synthetic pesticides or fungicides. For this reason, this text discusses the bioactivity potential of key chemotypic marker compounds (Section 6.10), based on documented biological effects. This underlines their pharmacological relevance and helps guide future research efforts. Such a chemotype-oriented approach may also support the rational selection of plant material for specific end uses, facilitating the development of standardized PFEO-based products with defined chemical and biological profiles.

In addition to the major constituents commonly found in different chemotypes of PF, some compounds may represent secondary chemotypic markers and contribute to observed bioactivities. For instance, a study showed that one of the trimethylbenzaldehyde isomers exhibited considerably lower antimicrobial activity than *α*-pinene and sabinene [204]. In addition, the EOs of many species within the genus *Ferula* L., which is closely related to *Prangos* Lindl., are characterized by significant amounts of trimethylbenzaldehyde isomers, often accompanied by significant levels of chrysanthenyl acetate and/or *β*-ocimene [121]. A wealth of scientific evidence supports the biological activities associated with these species [205]. Given that a similar chemical profile has also been observed in certain populations of PF, further targeted research in this direction could be proposed. Moreover, certain rare PF samples showed notable deviations in their chemical profiles, marked by the dominance of some alternative compounds. For instance, a root-specific oil sample from Montenegro predominantly contained 3,5-nonadiyne, reported to inhibit NO production in macrophages in a dose-dependent manner, without exhibiting any toxicity in rats [41]. Some studies have documented elevated levels of linalool [49] and limonene [64,79], which are well known for their broad-spectrum biological activities, including neuroactive, anti-inflammatory, antioxidant, and antimicrobial properties [131,206,207]. *β*-Elemene, identified as a predominant compound in certain studies [34], is widely recognized for its potent antitumor properties [208]. Although less frequent, these variations may carry pharmacological relevance and should be considered in future investigations, particularly in the context of plant application and the rational design of plant-based therapeutics.

Overall, the observed chemical diversity and bioactivity patterns indicate that PFEO represents a promising but still underexplored natural resource, with potential applications spanning pharmaceuticals, agriculture, and plant-based bioactive formulations. It is worth noting that although *α*-himachalene was initially reported as one of the major sesquiterpene constituents in PFEO [61], subsequent investigations have not confirmed its presence in appreciable amounts. Despite its relatively limited natural occurrence, being primarily associated with conifers, especially those in the *Cedrus* Trew genus (Pinaceae), this bicyclic hydrocarbon demonstrates notable pharmacological potential, including antiepileptic, antidepressant, insecticidal, and spasmolytic effects [209]. Given the outdated nature of that record and the lack of follow-up phytochemical studies related to Armenia (from where the plant material was originally collected), a renewed chemical profiling of this population could be of scientific value, potentially uncovering unique chemotypes with promising bioactive properties.

## Figures and Tables

**Table 1:** Summary of *Prangos ferulacea* (L.) Lindl. Essential Oil (PFEO) Composition from Various Plant Organs.

Plant Organ (Part)	Plant Material Origin	the Main EO Constituents (≥3%)	Reference ^c^
root ^a^	Iran: West Azerbaijan Province	*β*-phellandrene (32.1%), *m*-tolualdehyde (3-methylbenzaldehyde; 26.2%), *δ*-3-carene (25.8%), *α*-pinene (4.7%)	[14]
	Iran: Kohgiluyeh & Boyer-Ahmad Province	*δ*-3-carene (22.5%), *β*-phellandrene (11.8%), *α*-pinene (8.6%), terpinolene (7.2%), *p*-cymene (6.3%), *α*-phellandrene (6.2%), myrcene (4.5%), sabinene (3.6%), bornyl acetate (3.2%), *γ*-terpinene (3%)	[50]
	Montenegro: * Central Region	3,5-nonadiyne (85%)	[41]
stem ^a^	Iran: Province of Esfahan	1,8-cineole (19%), *α*-pinene (10.3%), caryophyllene oxide (4.2%), linalool (3.7%)	[49]
	Iran: *** North Khorasan Province	*α*-phellandrene (40%), *α*-pinene (14.8%), *β*-phellandrene (8%), (*Z*)-*β*-ocimene (7.5%), limonene (6.9%), *o*-cymene (4.9%), myrcene (3.6%)	[63]
leaf ^a^	Iran: Province of Esfahan	linalool (36.7%), caryophyllene oxide (16.3%), *α*-pinene (12.1%), 1,8-cineole (8.9%)	[49]
	Iran: Province of Kordestan	*β*-pinene (29.6%), *α*-pinene (19.8%), *δ*-3-carene (11.4%), *β*-phellandrene (11.1%), *β*-caryophyllene (3.7%)	[55,64]
	Iran: Khuzestan Province	*β*-pinene (9.4–27.9%), *δ*-3-carene (8.1–20.6%), *α*-pinene (4.8–16.4%), viridene (2.1–7.6%), terpinolene (1.7–6.8%), (*Z*)-nerolidol (1–9.2%), *β*-phellandrene (0.9–13.1%), *α*-bisabolol (0.9–10.2%), germacrene B (0.2–8.6%), caryophyllene oxide (nd–7.8%), *α*-phellandrene (nd–6.1%), (*Z*)-ternine (nd–5%), (2*Z*,6*Z*)-farnesol (nd–3.5%), *γ*-muurolene (nd–3.3%)	[52]
	Iran: ** Khuzestan Province	*δ*-3-carene (9.7%), *α*-bisabolol (8.2%), (*Z*)-ternine (8.2%), viridene (8.1%), *β*-pinene (7.7%), *α*-pinene (6.6%), germacrene B (6.2%), caryophyllene oxide (6%), (*Z*)-nerolidol (4.2%), *γ*-muurolene (3.2%)	[52]
	Iran: Fars Province	*α*-pinene (36.8%), camphene (15.8%), limonene (10.5%), *β*-pinene (8.7%), myrcene (5.9%), bornyl acetate (5.2%)	[56]
	Iran: ** Fars Province	(*E*)-*β*-ocimene (13.6%), 2,3,6-trimethylbenzaldehyde (12.7%), *p*-cymene (9.7%), terpinolene (8.3%), *β*-caryophyllene (5.4%), *β*-elemene (5.3%), germacrene D (5%), *α*-bisabolol (4.9%), limonene (4.5%), kessane (3.4%), *γ*-terpinene (3.4%), *α*-pinene (3%)	[53]
	Iran: ** Fars Province	(*E*)-*β*-ocimene (28.2%), limonene (12.2%), terpinolene (8.7%), *p*-cymene (7.1%), 2,3,6-trimethylbenzaldehyde (7%), germacrene D (5%), *β*-caryophyllene (3.9%), *α*-pinene (3.7%), *β*-elemene (3.5%), *γ*-terpinene (3.3%)	[54]
	Iran: ** Fars Province	(*E*)-*β*-ocimene (22.1%), limonene (15.2%), 2,3,6-trimethylbenzaldehyde (8.6%), *p*-cymene (7.6%), terpinolene (6.6%), (*E*)-caryophyllene (4.4%), *β*-elemene (3.7%), *α*-pinene (3.2%), *α*-bisabolol (3%)	[54] ^1^
	Iran: ** Fars Province	(*E*)-*β*-ocimene (23.6%), limonene (13.3%), *p*-cymene (12.2%), 2,3,6-trimethylbenzaldehyde (7.4%), terpinolene (6.7%), *α*-pinene (3.9%), *β*-caryophyllene (3.1%)	[53] ^2^
	Iran: *** North Khorasan Province	*α*-phellandrene (30%), *α*-pinene (17.1%), *p*-cymene (8.2%), limonene (7.6%), *β*-phellandrene (6.6%)	[63]
	Türkiye: *** Bitlis Province	*β*-elemene (48.9%), *α*-terpinolene (14.6%), ethylbenzene (9.7%), anisyl acetone (5.3%), 1-isopropyl-5-methylbicyclo[3.2.2]non-3-en-2-one (4.2%), *p*-xylene (3.4%)	[34]
	Italy: Palermo Province, Sicily	(*Z*)-*β*-ocimene (64.9%), sabinene (10.1%), *β*-caryophyllene (7.6%), (*E*)-*β*-ocimene (4.6%)	[51]
flower	Iran: East Azerbaijan Province	*α*-pinene (42.2%), (*Z*)-ocimene (stereoisomer not specified) (36.3%), myrcene (5%), *β*-phellandrene (3.3%)	[60]
	Iran: Fars Province	*α*-pinene (20.9%), bornyl acetate (13.8%), camphene (11.9%), limonene (8.6%), *β*-pinene (7.5%), myrcene (6%), *β*-caryophyllene (3.9%)	[56]
	Iran: Province of Esfahan	linalool (19%), lavandulyl acetate (16%), 1,8-cineole (14.5%), *α*-pinene (12.4%), geranyl isobutyrate (12.2%), *α*-campholenal (7%), *α*-cadinol (6.4%)	[49]
	Iran: Khuzestan Province	*β*-pinene (18.6%), *α*-pinene (12.5%), *δ*-3-carene (12.2%), viridene (8%), (*Z*)-nerolidol (6.3%), *α*-bisabolol (4%), *β*-phellandrene (3.1%)	[52]
	Iran: Province of Kordestan	*β*-pinene (20.6%), *δ*-3-carene (10.4%), *α*-pinene (8.8%), *β*-phellandrene (8.1%), germacrene D (5.9%), *α*-humulene (5.3%), *p*-cymene (3.8%), *δ*-cadinene (3.3%)	[55,64]
	Italy: Palermo Province, Sicily	(*Z*)-*β*-ocimene (44.4%), sabinene (20.2%), *γ*-terpinene (8.1%), 3-thujene (5.8%), *α*-pinene (4.3%), limonene (3.3%), *α*-terpinene (3%)	[51]
fruit	Iran: East Azerbaijan Province	*α*-pinene (63.1%), (*Z*)-ocimene (stereoisomer not specified) (9.7%), *β*-pinene (8.3%), myrcene (4.8%)	[60]
	Iran: Tehran Province	*β*-pinene (33%), *α*-pinene (10.1%), *δ*-3-carene (10%), limonene (8.9%), germacrene D (5.3%)	[47]
	Iran: Tehran Province	chrysanthenyl acetate (26.5%), limonene (19.6%), *α*-pinene (19.5%), *δ*-3-carene (6.6%), 2,4,6-trimethylbenzaldehyde (mesitaldehyde; 6.1%), germacrene B (3.5%)	[65]
	Iran: Khuzestan Province	*β*-pinene (26.8%), *α*-pinene (18%), *δ*-3-carene (12.1%), *β*-phellandrene (8.9%), (*Z*)-nerolidol (3.2%)	[52]
	Iran: ^b^ Khuzestan Province	*δ*-3-carene (20.5%), *β*-pinene (20.2%), *β*-phellandrene (12.9%), *α*-pinene (11.1%), viridene (8.3%), terpinolene (7.3%), *α*-phellandrene (7%)	[52]
	Iran: Isfahan Province	limonene (17.8%), *α*-pinene (6.8%), *β*-pinene (5.9%), *α*-bisabolol (5.1%), *β*-phellandrene (4.9%)	[66]
	Iran: North Khorasan Province	*α*-phellandrene (47.2%), *β*-phellandrene (13.4%), *α*-pinene (11.8%), limonene (6.5%), *β*-ocimene (*E/Z* configuration not specified) (5.4%), *p*-cymene (5.6%), myrcene (3.6%)	[63]
	Türkiye: Kastamonu Province	*γ*-terpinene (30.2–33.3%), *α*-pinene (12.8–16.7%), *p*-cymene (9.8–10.8%), (*E*)-*β*-ocimene (7.7–8%), (*Z*)-*β*-ocimene (6.4–7.1%), germacrene B (2.6–6.6%), *β*-phellandrene (2.9–3.2%)	[57]
	Türkiye: Bitlis Province	*β*-elemene (26.9%), *β*-phellandrene (18.9%), *α*-terpinolene (14.2%), *α*-phellandrene (9.7%), *p*-cymene (5.1%), *α*-pinene (4.1%)	[34]
	Türkiye: Tunceli Province	2,3,6-trimethylbenzaldehyde (66.6%), chrysanthenyl acetate (15.1%), *β*-ocimene (*E*/*Z* configuration not specified) (3.8%), *p*-mentha-1,5-dien-8-ol (3.6%)	[58]
	Italy: Sassari Province, Sardinia	*α*-pinene (18.2%), sabinene (15.9%), limonene (15.1%), *cis*-chrysanthenyl acetate (14.5%), 2,3,4-trimethylbenzaldehyde (13%), *γ*-terpinene (3.3%), *p*-cymene (3%)	[29]
	Italy: Perugia Province, Umbria	*γ*-terpinene (27.8%), (*Z*)-*β*-ocimene (26.8%), terpinen-4-ol (12.2%), (*E*)-*β*-ocimene (7.8%), *p*-cymene (6.9%), *α*-pinene (4.1%)	[59]

^a^ collected during the flowering period, except specimens marked with * (fruiting period), ** (pre-reproductive period) and *** (not specified); ^b^ immature fruits; ^c^ all samples were obtained by hydrodistillation, except: ^1^ hydrodistillation with ultrasound pretreatment and ^2^ ohmic-assisted hydrodistillation; nd: not detected.

**Table 2:** Summary of PFEO Constituents in Aerial Parts.

Phenological Phase	Plant Material Origin ^a^	the Main EO Constituents (≥3%)	Reference ^d^
pre-reproductive	Iran: * Fars Province	terpinolene (56.3%), *γ*-terpinene (6.1%), *β*-phellandrene (5.8%), isopentyl isovalerate (4.7%), *β*-caryophyllene (4.7%), bornyl acetate (3%)	[46]
	Iran: * Fars Province	*δ*-3-carene (45.9%), indole (11.6%), terpinolene (9.6%), *p*-cymen-8-ol (6.2%), *n*-pentadecanol (5.5%)	[67]
	Iran: Fars Province	terpinolene (38.1%), *γ*-terpinene (13.2%), *α*-phellandrene (6.2%), *trans*-chrysanthenyl acetate (4%), (*Z*)-*β*-ocimene (3.5%), *β*-caryophyllene (3.6%)	[46]
	Iran: Fars Province	limonene (55.1%), *γ*-terpinene (10.7%), bornyl acetate (8.5%)	[67]
	Iran: ** East Azerbaijan Province	*α*-pinene (57.0%), 3-ethylidene-2-methyl-1-hexen-4-yne (5.3%), *β*-pinene (4.5%), (*E*)-anethole (3.9%), caryophyllene oxide (3.5%)	[45]
	Iran: Lorestan Province	*β*-pinene (43%), *α*-pinene (40%), *β*-phellandrene (6.5%), *α*-terpinene (5.1%)	[68]
	Iran: Kermanshah Province	*β*-caryophyllene (33.1%), caryophyllene-oxide (24.7%), *α*-humulene (8.3%), *p*-cymene (3.4%)	[48]
	Iran: Semnan Province	*β*-phellandrene (20.4%), *α*-terpinolene (15.3%), *α*-pinene (11.6%), *δ*-3-carene (11.1%), (*E*)-*β*-ocimene (9.7%), *α*-phellandrene (9.1%), myrcene (4.5%), sabinene (4.4%), *γ*-terpinene (3.4%)	[69]
	Greece: * Lasithi, Crete	*γ*-terpinene (27.5%), *β*-phellandrene (16.3%), *α*-pinene (10.4%), *α*-terpinolene (9%), (*E*)-*β*-ocimene (8.8%), *p*-cymene (6.8%), apiole (5.5%), myrcene (4.4%)	[70]
flowering	Iran: East Azerbaijan Province	(*E*)-anethole (95.5)	[45]
	Iran: West Azerbaijan Province	*β*-pinene (43.1%), *α*-pinene (22.1%), *δ*-3-carene (16.9%), *α*-terpinolene (3.9%)	[71]
	Iran: * Kohgiluyeh and Boyer-Ahmad Province	*β*-pinene (27%), *δ*-3-carene (24. 8%), *α*-pinene (18.3%), *β*-caryophyllene (17.7%)	[72]
	Iran: * Fars Provinc	*α*-pinene (41.4%), *δ*-3-carene (34.6%), limonene (14.6%), *β*-pinene (9.5%), terpinolene (8.1%), myrcene (7.4%), sabinene (4.7%), *α*-phellandrene (4.1%)	[67]
	Iran: Fars Province	*α*-pinene (24.2%), *β*-pinene (8.6%), *δ*-3-carene (7.7%), terpinolene (3.8%), *β*-phellandrene (4.4%)	[67]
	Iran: ^b^ Kermanshah Province	*β*-caryophyllene (42.5%), *α*-humulene (8.9%), spathulenol (9.4%), *α*-bisabolol (4.4%), *p*-cymene (3.3%), linalool (3.2%), *δ*-3-carene (3%)	[48]
	Iran: ^c^ Kermanshah Province	*β*-caryophyllene (48.2%), *α*-humulene (10.3%), spathulenol (6.7%), *α*-bisabolol (4.2%), *δ*-3-carene (3.4%), linalool (3.5%), (*E*)-*β*-farnesene (3.2%)	[48]
	Iran: ^c^ Kermanshah Province	*β*-caryophyllene (52.3%), spathulenol (10.4%), *α*-bisabolol (5.3%), (*E*)-*β*-farnesene (5%), *α*-humulene (4%), *β*-eudesmol (3.5%)	[48] ^1^
	Iran: ** Kermanshah Province	*β*-caryophyllene (48.9%), spathulenol (9.7%), *α*-humulene (4.05%), *α*-bisabolol (3.3%), (*E*)-*β*-farnesene (3.1%)	[73] ^1^
	Iran: ** Kermanshah Province	*β*-caryophyllene (54.5–60.4%), spathulenol (4.8–11.4%), *α*-humulene (4.6–4.8%), *α*-bisabolol (2.9–3.6%), (*E*-*β*-farnesene (0.9–3.2%), *δ*-3-carene (0.5–3%), dihydrocarveol acetate (0.2–6%)	[73] ^2^
	Iran: Lorestan Province	*α*-pinene (37.1%), *β*-pinene (33.8%), *δ*-3-carene (6.7%), *α*-terpinene (6.5%), *β*-phellandrene (5.6%), terpinolene (4.9%)	[68]
	Iran: Lorestan Province	*α*-pinene (36.6%), *β*-pinene (31.9%), *β*-phellandrene (11.7%), *α*-terpinolene (6.9%), *α*-phellandrene (3.9%), *β*-caryophyllene (3.1%)	[74]
	Italy: * Palermo Province, Sicily	(*E*)*-β*-ocimene (43.1%), (*Z*)-*β*-ocimene (15.8%), *α*-pinene (5.6%), carvacrol (3.6%)	[75]
fruiting	Iran: Lorestan Province	*α*-pinene (31.7%), *β*-pinene (38.5%), *β*-phellandrene (10.3%), terpinolene (5.1%), *α*-terpinene (4.9%), *p*-cymene (3.2%)	[68]
	Iran: ^b^ Kermanshah Province	spathulenol (32.9%), *β*-caryophyllene (27%), (*E*)-*β*-farnesene (5.8%), *δ*-3-carene (6.8%), limonene (4.3%), *α*-humulene (3.3%)	[48]
	Iran: ^c^ Kermanshah Province	spathulenol (35.2%), *β*-caryophyllene (32.1%), (*E*)-*β*-farnesene (13%)	[48]
	Iran: Kurdistan province	*β*-pinene (22.9%), *δ*-3-carene (16%), *α*-pinene (12.6%), epi-*α*-bisabolol (7.7%), terpinolene (3.5%), limonene (3.1%)	[47] ^1^
not specified	not specified	*β*-pinene (27%), *δ*-3-carene (24.9%), *α*-pinene (18.3%), sabinene (17.7%)	[76]
	Türkiye: local market	*β*-phellandrene (22.3%), *α*-pinene (16.2%), *p*-cymene (11.2%), *β*-myrcene (7.2%), indene (6.4%)	[33] ^3^

^a^ dried plant material used, except samples marked with * (fresh) and ** (not specified); ^b^ flowering individuals; ^c^ non-flowering individuals; ^d^ all samples were obtained by hydrodistillation, except: ^1^ steam distillation, ^2^ distillation via forage quality optimizer system and ^3^ headspace solid-phase microextraction.

**Table 3:** Antimicrobial activity of PFEO.

Microbial Group	Species	Strain	MIC µg/mL	DD IZ mm	WD IZ mm	Reference(s)
Gram-positive bacterium	*Staphylococcus aureus*	ATCC 6538	20	21.5	-	[14]
		ATCC 1112	0.5 ^a^, 2 ^b^, 0.5 ^c^	-	-	[49]
		ATCC 29737	6.5	14	-	[65]
		ATCC 6538P	>200 ^c^, >200 ^a^	-	-	[51]
		ATCC 25923	4300 ^d^, 4300 ^e^, 5500 ^f^	16.8 ^d^, 15.3 ^e^, 14.3 ^f^	-	[53,54]
		PTCC 1113	-	-	35	[74]
	*Staphylococcus epidermidis*	ATCC 12228	20	19	-	[14]
		ATCC 1114	0.25 ^a^, 0.5 ^b^, >4 ^c^	-	-	[49]
		ATCC 14990	6.8	13	-	[65]
		PTCC 1349	-	-	22	[74]
		PTCC 1114	-	9–12 ^g^	-	[60]
	*Staphylococcus saprophyticus*	PTCC 1379	-	-	14	[74]
		PTCC 1113	8.19 ^h^	-	6.6–11 ^i^	[69]
	*Bacillus cereus*	ATCC 10876	-	15	-	[60]
		ATCC 10987	100 ^c^, 100 ^a^	-	-	[51]
		ATCC 11778	6200 ^d^, 6200 ^e^, 6200 ^f^	14.7 ^d^, 15 ^e^, 11.7 ^f^	-	[53,54]
		ATCC 1015	1 ^a^, 2 ^b^, 0.5 ^c^	-	-	[49]
		clinical isolate	na	-	na	[69]
	*Bacillus subtilis*	ATCC 6633	11	7	-	[65]
		AZ59	100 ^c^, 100 ^a^	-	-	[51]
	*Enterococcus faecalis*	ATCC 51299	2.27	-	23	[110]
	*Listeria inocua*	ATCC 33090	6200 ^d^, 12,500 ^e^, 12,500 ^f^	15.3 ^d^, 12.5 ^e^, 10.7 ^f^	-	[53,54]
Gram-negative bacterium	*Escherichia coli*	ATCC 8739	5	14	-	[14]
			12.5	12		[65]
		ATCC 15224	12,500 ^d^, 12,500 ^e^, 25,000 ^f^	11.3 ^d^, 13.3 ^e^, 11.3 ^f^	-	[53,54]
		PTCC 1330	3.27 ^h^	-	6.2–14 ^i^	[69]
					28	[74]
		PTCC 1047	-	9–12 ^g^	-	[60]
		DH5α	>200 ^c^, >200 ^a^	-	-	[51]
	*Pseudomonas aeruginosa*	ATCC 9027	20	12	-	[14]
			10	5		[65]
		ATCC 1310	0.06 ^a^, 0.5 ^b^, 1 ^c^	-	-	[49]
		ATCC 27853	-	9–12 ^g^	-	[60]
		PTCC 8	-	-	8	[74]
		PAOI	>200 ^c^, >200 ^a^	-	-	[51]
	*Salmonella paratyphi*	ATCC 4420	10	14.5	-	[14]
	*Salmonella typhi*	PTCC 1185	-	-	25	[74]
	*Salmonella typhimurium*	ATCC 202026	25,000 ^d^, 25,000 ^e^, 25,000 ^f^	10 ^d^, 10.8 ^e^, 9.3 ^f^	-	[53,54]
		ATCC 14028	100 ^c^, >200 ^a^	-	-	[51]
	*Shigella flexneri*	PTCC 1234	-	-	20	[74]
	*Klebsiella aerogenes*	ATCC 13048	25,000 ^d^, 25,000 ^e^, 25,000 ^f^	10.2 ^d^, 10.7 ^e^, 9.8 ^f^	-	[53,54]
Yiest	*Candida albicans*	ATCC 10231	5	14	-	[14]
		clinical isolate	0.0097–0.0195 ^j^	-	-	[72]
	*Candida parapsilosis*	clinical isolate	0.0097 ^j^	-	-	[72]
	*Candida glabrata*	clinical isolate	0.0097 ^j^	-	-	[72]
	*Candida krusei*	clinical isolate	0.0097 ^j^	-	-	[72]
	*Candida kefyr*	ATCC 38296	-	9–12 ^g^	-	[60]

^a^ leaf EO; ^b^ stem EO; ^c^ flower EO; ^d^ obtained by hydrodistillation; ^e^ obtained by hydrodistillation with ultrasound pretreatment; ^f^ obtained by ohmic-assisted hydrodistillation; ^g^ reported as a range in the original study; ^h^ MIC values were approximated based on the lowest concentration showing a visible IZ in agar well diffusion (should be interpreted as indicative only); ^i^ depending of the concentration used (from 8.19 to 800 µg/mL); ^j^ MIC range value (µL/mL) based on testing of *n* = 13 clinical isolates, except for *C. krusei* (*n* = 10); MIC = Minimal Inhibitory Concentration; DD = Disk Diffusion; WD = Well Diffusion; IZ = Inhibition Zone; na = not active.

## Data Availability

All data generated or analyzed during this study are included in this published article.
